# Engaging communities in the control of arboviral diseases: insights from the African region

**DOI:** 10.1371/journal.pntd.0013300

**Published:** 2025-07-15

**Authors:** Anankpo Gildas Yahouédo, Corinne S. Merle, Emmanuel Chanda, Ingrid B. Rabe, Florence Fouque, Diana P. Rojas, Abraham Aseffa, Christine M. Halleux, Raman Velayudhan

**Affiliations:** 1 UNICEF/UNDP/World Bank/WHO Special Programme for Research and Training in Tropical Diseases (TDR), World Health Organization, Geneva, Switzerland; 2 Global Malaria Programme, World Health Organization, Geneva, Switzerland; 3 Health Emergencies Programme, World Health Organization, Geneva, Switzerland; 4 Department of Control of Neglected Tropical Diseases, World Health Organization, Geneva, Switzerland; Gdanski Uniwersytet Medyczny, POLAND

## Introduction

Arboviral diseases, transmitted by vectors such as mosquitoes and ticks, continue to pose increasing public health threats in Africa [1] and all other continents. Factors contributing to increase include unplanned urbanization, population growth, malnutrition, poor waste management, inadequate water treatment, cross-border mobility, and social inequalities [2,3]. Controlling arboviral diseases is complex due to the intricate interactions among pathogens, vectors, hosts, and environmental factors, requiring integrated approaches [4]. Anticipated increases in temperature may increase water shortages, leading to water hoarding and creating more mosquito aquatic habitats [4]. Community engagement is a strategic approach in the pillars of the Global Arbovirus Initiative, and has been recognized as a key component in the successful implementation of arboviral disease control programmes [5]. Active engagement of communities requires a shift from traditional top-down interventions to approaches that incorporate local knowledge and perspectives [6]. Engaging communities in the prevention and control of arboviral diseases should cultivate a sense of ownership and responsibility for vector control efforts, thereby enhancing compliance with preventive measures and promoting sustained behavioral change. However, there are obstacles to effective engagement of communities in arboviral disease prevention and control efforts [7].

Based on responses to a region-wide survey, this viewpoint summarizes some of the barriers to community sensitization and engagement in activities related to the control of arboviral diseases across all 47 countries in the World Health Organization (WHO) African Region. Community sensitization educates and raises awareness, whereas engagement promotes collaboration by involving communities in decision-making, planning, implementation, and evaluation of interventions. The survey was initiated to guide countries as part of the preparedness and response efforts to the growing outbreaks/epidemics of arboviral diseases in the region [8]. Countries were assessed in 2021 through an online self-administered questionnaire, hosted on WHO’s platform, covering five capacity indicators and other barriers for community sensitization and engagement in the control of arboviral diseases. The capacity indicators included (i) existence of outreach programmes for conducting community sensitization and engagement activities, (ii) their effective functioning, (iii) regular training of outreach programme staff, (iv) optimal geographical coverage (across all districts), and (v) sufficient financial resources for the functioning of outreach programmes. All 47 countries (100%) submitted their responses. The respondents were national experts from ministries of health, national health departments, and research institutes. Since the barriers were reported using a self-administered questionnaire, there might be potential for bias, implying that they should be considered only indicative.

In order to provide a broader perspective than the data gathered in the survey alone, additional key barriers were explored in pertinent literature. Barriers reported in both the survey and the literature are discussed to propose a more cohesive way forward for enhancing community sensitization and engagement.

## Barriers to effective community sensitization and engagement in the control of arboviral diseases in the African region

### Barriers identified through a survey

**Fig 1:**
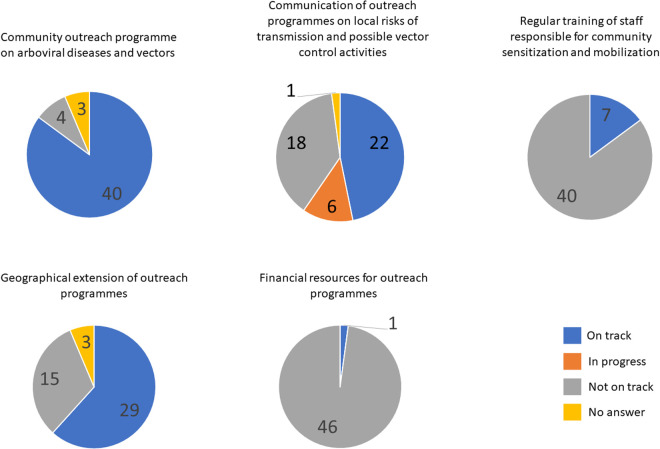
Capacity indicators related to community sensitization and engagement for the prevention and control of arboviral diseases in the WHO African Region.

The survey identified three critical barriers: (i) lack of financial resources to cover staff time and activities of outreach programmes (reported by 46/47 countries, 98%), (ii) lack of training of community healthcare workers, community leaders, and heads of community-based organizations responsible for community sensitization and mobilization (reported by 40/47 countries, 85%), and (iii) lack of adequate communication with communities on the risk of transmission of arboviral diseases and vector control activities (reported by 24/47 countries, 51%) (Fig 1). Despite the existence of outreach programmes in 85% of countries, they do not effectively achieve the objectives due to the identified barriers. Other challenges included: the lack of educational and sensitization materials for both community trainers and healthcare workers, message misinterpretation, insufficient community health workers, the absence of national guidelines for outreach programmes, and insecurity in remote areas [8].

### Barriers identified from the literature

To gain a comprehensive understanding of barriers, the literature was reviewed to identify additional key barriers not mentioned in the survey but pertinent to the African region. Indeed, community engagement lacks standardized methods across diverse cultural and socioeconomic contexts [9]. The variability of communities and arboviral diseases requires customized approaches, making universal strategies ineffective.

Community-based interventions struggle with long-term sustainability and scalability, especially in resource-limited settings. Despite small-scale studies showing effectiveness, maintaining impact over time and expanding to larger populations remains challenging due to competing health priorities and limited funding [10].

There is limited integration of community engagement approaches into existing national strategies for the prevention and control of arboviral diseases [10]. The siloed nature of many interventions can lead to duplication of efforts and inefficient use of resources.

Communicating complex medical and scientific information poses challenges in community engagement due to technical intricacies, evolving research, and expert disagreements [11]. Local communication difficulties often require escalation through social and political hierarchies [9]. Additional obstacles include a lack of transparency, limited multilingual materials, and unsustainable communication tools [9].

The use of digital tools and mobile technologies for community engagement in arboviral disease control is underexplored despite their potential in improving disease surveillance and health communication [12].

## Way forward

Considering the barriers reported in both the survey and the literature, successful engagement of communities in the control of arboviral diseases in the African Region requires multisectoral collaboration and the identification and implementation of long-term solutions that prioritize the ideas of affected communities. Knowledge, attitude, and practices surveys are needed to identify these ideas but also opportunities to improve community sensitization and engagement. Implementation research, policy, and practice are needed to inform strategies for overcoming the barriers. Table 1 provides a summary of the barriers and suggested solutions, including: increased funding, community capacity building, tailored information, education and communication (IEC) materials, integration of volunteer community members into the primary healthcare system, trust-building and enhanced communication, sustained community engagement, use of digital tools by communities, and addressing insecurity and safety concerns impeding community involvement or identifying alternatives approaches when insecurity is a persistent challenge. Policymakers and national disease control programmes in the African Region are encouraged to consider implementing these solutions, which are also applicable to other diseases.

**Table 1:** Barriers and suggested solutions for disease control programmes.

Barrier	Suggested solutions
**Lack of financial resources**	Increase funding allocation from government, municipality, private sector, and international organizations Leverage public-private partnerships Explore grants and donations from non-governmental organizations (NGOs) and philanthropic foundations Promote community fundraising Promote innovation and innovative cost-saving approaches
**Training gaps among community stakeholders**	Develop a training plan, method, and frequency that prioritizes community needs and allocates the necessary budget
**Lack of educational and sensitization materials**	Develop tailored IEC materials pre-tested with representatives of target audiences. Refer to the Risk Communication and Community Engagement Action Plan guidance developed by WHO amid the COVID-19 pandemic [13]
**Communication-related aspects with communities**	Refer to WHO guidelines on risk communication and community engagement readiness and response toolkit [14]
**Lack of national guidelines integrating community engagement strategies**	Develop a national guideline for arboviral disease control by adapting WHO recommendations [14,15], focusing on collaboration between community initiatives, local health authorities, and national control programmes
**Long-term community engagement for sustainability and scalability of community-based interventions**	Establish multisectoral collaboration for a more effective use of resources, to bring new allies onboard, and improve funding Improve the health outcomes of affected communities for trustful lasting collaborations Smoothen communication across community groups Build community trust through high standards of ethical conduct throughout engagement Ensure that skilled career staff lead community engagement processes to sustain trust and partnership among community groups Integrate community-based interventions into existing local initiatives
**Limited use of digital tools (intuitive apps and platforms) and mobile technologies for community engagement in vector control activities**	Explore the opportunity and feasibility to use available user-friendly tools for engaging communities in vector control activities. EWARS in a box by WHO can be adapted to include arboviruses Conduct awareness campaigns to educate the community about the benefits of using digital tools for vector control Build capacity of community health workers (CHW) and local stakeholders on the use of digital tools Collaborate with technology companies, NGOs to provide the necessary resources and expertise to overcome challenges related to digital tool usage
**Inaccessibility to remote areas for insecurity reasons**	Work with local security forces and community leaders to ensure that health workers and community members can operate safely Consider promoting the use of digital tools and mobile technologies in these remote areas

## Conclusion

Community engagement is a key approach for controlling arboviral diseases in the African Region, but important barriers must be addressed through multisectoral collaboration ranging from policymakers to community members. Strategies for community engagement should be evidence-informed, culturally appropriate, and sustainable. Existing resources (guidelines, tools) can help African authorities and communities apply effective engagement approaches, potentially reducing arboviral disease threats when combined with existing and innovative vector control methods.
